# Early Pediatric Fractures in a Universally Insured Population within the United States

**DOI:** 10.1186/s12887-019-1725-y

**Published:** 2019-10-08

**Authors:** Jared A. Wolfe, Heather Wolfe, Amanda Banaag, Scott Tintle, Tracey Perez Koehlmoos

**Affiliations:** 1 Department of Orthopaedics, Walter Reed National Military Medical Center, 8901 Rockville Pike, Bethesda, MD 20889 USA; 2 Department of Obstetrics and Gynecology, Malcolm Grow Medical Clinic, 1060 Perimeter Rd, Joint Base Andrews, Prince George’s County, MD 20762 USA; 3 Department of Health Services Administration, The Henry M. Jackson Foundation for the Advancement of Military Medicine, Inc., Bethesda, USA; 4 Department of Health Services Administration, Department of Preventive Medicine & Biostatistics, Uniformed Services University of the Health Sciences, Bethesda, USA

**Keywords:** Pediatric fractures, Military health system, United States, Child health, Injury, Osteogenesis imperfecta, Child abuse

## Abstract

**Background:**

Musculoskeletal injury, including fracture, is one of the most common causes of morbidity in pediatric patients. The purpose of this epidemiologic study is to determine the prevalence and risk factors for fracture in a large cohort of pediatric patients under the age of 5.

**Results:**

Of the 233,869 patients included in the study, 13,698 fractures were identified in 10,889 patients. The highest annual incidence was in the 4 year old age group with a rate of 24.2 fractures per 1000 children. The annual incidence within all age groups was 11.7 fractures per 1000 children. The two most common fractures were forearm and humerus fractures. Fracture incidence was increased in male children, patients who live outside the US, and in Caucasian patients. An increase in rate of fracture was also identified in children of officers when compared with children of enlisted service members. There were 35 abuse related fractures in our cohort, with 19 of them occurring in children less than 1 year old. Only three children in our cohort had Osteogenesis Imperfecta.

**Conclusion:**

Fractures are common injuries in young children with an incidence over the first 5 years of life of 5.86%. Multiple risk factors were also identified including age, race, geographic location and socioeconomic status. The results of this study are an important contribution to epidemiologic and public health literature and serve to characterize the incidence of and risk factors for sustaining an early childhood fracture.

## Background

Accidental injury is the leading cause of morbidity and mortality among children in the United States. It is estimated that 1 out of 4 children will sustain an accidental injury during childhood which will require urgent medical care [1]. This accounts for roughly 9.2 million visits to the emergency room per year. Approximately 15% of these patients will have a fracture [1]. In addition to the multitude of fractures presenting to the ER some children may present directly to an urgent care facility or primary care provider. Despite this large burden of disease, exact fracture incidence and characteristics including demographics and risk factors have not been well described.

The annual incidence of childhood fracture varies greatly across studies from about 12 fractures per 1000 children to 36.1 fractures per 1000 children with different factors suggested to impact the incidence including geographic location, age, and gender of the patient [2, 3]. Multiple methods of data collection, ranging from surveys to various databases and other methods of population based sampling, further increase this variability. Although existing studies attempt to estimate fracture incidence, many have significant limitations [4]. For example, these studies often estimate the population of the given area and assume that all fracture care is provided at the local hospital. These techniques reduce the precision of their calculated fracture rate. Additionally, many of these studies take place outside of the United States. Since geographic location has been shown to impact fracture incidence these results may not be applicable to the US population. Studies done within the US often limit the scope to inpatient or emergency department fractures only. As this technique does not account for fractures treated in the outpatient setting, this underestimates the true incidence of pediatric fracture. The purpose of this epidemiologic study is to use a large cohort of pediatric patients followed from birth until the age of 5 in order to determine the incidence of various extremity fractures as well as to further characterize demographics and identify risk factors.

## Methods

The Military Health System Data Repository (MDR) is the administrative claims data set for all health service utilization and pharmaceutical claims by active duty service members in the US (Army, Navy, Air Force, Marines), retirees, and their dependent beneficiaries. TRICARE beneficiaries have the unique option of receiving care at a military treatment facility (direct care) or a civilian medical facility (purchased care). The Military Health System is separate from the care delivered through the Veterans Administration. The MDR does not include care received in a combat zone. All care, whether direct or purchased is captured by the MDR [5–8].

The MDR was initially queried for all infants born between 2006 and 2009, and then evaluated by using the Defense Enrollment Eligibility Reporting System (DEERS) in order to ensure a minimum of 5 years of medical beneficiary eligibility. The included population’s data were then searched for any ICD-9 code corresponding to an extremity fracture. All ICD-9 codes between 810 and 829 were included (Table 1). Fractures were recorded on the basis of ICD-9 code and age of occurrence. Once a specific ICD-9 code was identified for a patient any additional appearance of the code for the next year was considered to be related to that initial injury and was not recorded. After 1 year it was attributed to a new fracture and was recorded again. MDR was also queried for every patient with a fracture for ICD-9 code 995.59 for child abuse and neglect. If the fracture and abuse occurred within 2 weeks of each other the fracture was considered to have been related to abuse. MDR was further queried for ICD-9 code 756.51 to determine the presence of Osteogenesis Imperfecta in our fracture population.

**Table 1:** List of ICD-9 codes for fractures included in study

ICD-9 Code	Diagnosis
810	Fracture of the Clavicle
811	Fracture of the Scapula
812	Fracture of the Humerus
813	Fracture of the Radius/Ulna
814	Fracture of the Carpal Bones
815	Fracture of the Metacarpal Bones
816	Fracture of the Phalanges of the Hand
817	One or More Hand Fractures
818	Ill-defined fracture of the Upper Extremity
819	Multiple fractures of Both Upper Extremities
820	Fracture of the Neck of the Femur
821	Fracture of other part of the Femur
822	Fracture of the Patella
823	Fracture of the Tibia/Fibula
824	Fracture of the Ankle
825	Fracture of one or more Tarsal/Metatarsal bones
826	Fracture of One or more phalanges of the foot
827	ill-defined fracture of the lower extremity
828	Multiple fractures of both the lower and upper extremities
829	Fracture of unspecified bone

Demographic data is also recorded for all patients, and elements such as gender, age at time of fracture, geographic location, rank of sponsor, and sponsor’s race were selected to determine potential influence on likelihood of fracture. This project was considered exempt by the Uniformed Services University of the Health Sciences Institutional Review Board.

## Statistical analysis

Calculations were performed on SAS software version 9.4 (SAS institute, Cary, North Carolina). Annual incidence was calculated in a rate of x per 1000 children and was further broken down into groups based on the year of occurrence. Logistic regression analysis was used to calculate the odds ratios with 95% confidence intervals for each of the designated risk factor categories: gender, geographic location, race, and rank of sponsor. Comparisons of categorical data were then made using a chi-squared test with *p*-value <.05 determined to be statistically significant.

## Results

Our study cohort consisted of 233,869 patients who were followed for 5 consecutive years. There were a total of 13,698 fractures in 10,889 children. The overall incidence of sustaining a fracture for the study time period was 5.86%. The annual incidence in each age group was 3.6 fractures per 1000 children (Age < 1), 9.8 fractures per 1000 children (Age 1), 10.7 fractures per 1000 children (Age 2), 10.2 fractures per 1000 children (Age 3), and 24.2 fractures per 1000 children (Age 4). The most common fractures in all age groups were forearm fractures, which increased in annual incidence with the age of the child from .56 per 1000 children at age 1 to 8.56 per 1000 children at age 4. The top five most common fractures in each age group were identified (Table 2 and Table 3). The data set was further queried to determine the rate of abuse-related fractures and the age at which they occurred. In our cohort there were 35 fractures attributed to child abuse. This was most common in the youngest age group with 19 of the abuse-related fractures occurring before the age of one. (Table 2) There were a total of three children with Osteogenesis Imperfecta in the study group, none of which had fractures attributed to child abuse.

**Table 2:** Fracture incidence and occurrence by age group

Number of Fractures by Age Group and Diagnosis Code																						
Age Group	Fracture Dx Codes																					
	810	811	812	813	814	815	816	817	818	819	820	821	822	823	824	825	826	827	828	829	Total	Annual Incidence per 1000 Children
<1	129	2	89	131	9	8	47	0	11	1	23	124	0	120	62	13	9	14	7	46	845	3.613133848
1	206	3	262	570	45	27	182	1	30	0	19	92	0	459	191	83	47	24	0	56	2297	9.82173781
2	334	2	328	573	36	31	229	3	23	0	25	132	1	374	134	140	74	17	0	49	2505	10.7111246
3	263	0	383	672	55	35	234	0	39	0	15	85	0	245	103	139	68	21	1	32	2390	10.21939633
4	395	6	1142	2003	160	100	560	7	73	0	21	120	0	293	224	226	143	29	2	157	5661	24.20585884
Total	1327	13	2204	3949	305	201	1252	11	176	1	103	553	1	1491	714	601	341	105	10	340	13698	11.71425029

**Table 3:** Most common fractures by age group

Age group in years	Most common fractures (annual incidence per 1000 children)	Abuse related fractures
< 1	1. Forearm (.56)	19
	2. Clavicle (.55)	
	3. Femur (.53)	
	4. Tibia/Fibula (.51)	
	5. Humerus (.38)	
1	1. Forearm (2.44)	16
	2. Tibia/Fibula (1.96)	
	3. Humerus (1.12)	
	4. Clavicle (.88)	
	5. Ankle (.82)	
2	1. Forearm (2.45)	
	2. Tibia/Fibula (1.60)	
	3. Clavicle (1.43)	
	4. Humerus (1.40)	
	5. Finger (.98)	
3	1. Forearm (2.87)	
	2. Humerus (1.64)	
	3. Clavicle (1.12)	
	4. Tibia/Fibula (1.04)	
	5. Finger (1.00)	
4	1. Forearm (8.56)	
	2. Humerus (4.88)	
	3. Finger (2.39)	
	4. Clavicle (1.69)	
	5. Tibia/Fibula (1.25)	

Males were more likely to sustain fractures than females with an increased fracture incidence of about 12% across the study population (*p* < .001). Patients located outside the continental United States were also more likely to sustain a fracture with an incidence about 40% higher than patients within the United States (*p* < .001). Among regions within the continental US, patients in the north region had the highest fracture rate, however differences between the north, south, and west were not statistically significant (Table 4, *p* = .326). Children of officers were 25% more likely to sustain a fracture than those of enlisted personnel (*p* < .001). Caucasian children had the highest fracture incidence compared to all other races except American Indian or Alaska Native (*p* < .001).

**Table 4:** Risk factors for fracture

	Odds Ratio	95% CI	*p*-value
Gender			
M	1.119	1.078–1.164	< 0.001
F (ref)	–	–	
Sponsor’s Race			
White (ref)	–	–	
Black	0.583	0.545–0.623	< 0.001
Asian or Pacific Islander	0.768	0.695–0.85	< 0.001
American Indian or Alaska Native	0.831	0.676–1.021	0.077
Other	0.795	0.701–0.901	< 0.001
Unknown	0.784	0.541–1.138	0.201
Tricare Region			
West (ref)	–	–	
North	1.062	1.011–1.116	0.017
South	1.026	0.975–1.081	0.326
OCONUS	1.448	1.35–1.553	< 0.001
Alaska	0.990	0.851–1.152	0.896
Sponsor’s Rank			
Junior Enlisted (ref)	–	–	
Senior Enlisted	1.131	1.082–1.183	< 0.001
Junior Officer	1.276	1.206–1.349	< 0.001
Senior Officer	1.228	1.028–1.466	0.024
Warrant Officer	1.234	1.027–1.481	0.025

## Discussion

The rate of fracture in pediatric patient populations has previously been shown to vary on the basis of age, gender, and geographic location, with most estimates placing the yearly incidence of fracture between 9 and 36 fractures per 1000 children [2, 3, 9, 10]. The variability in fracture incidence is likely related to country of origin of the article, method of data collection, and demographics of the patients included in the study. Our study found an annual fracture incidence of 11.7 fractures per 1000 children across all age groups, which is consistent with the previously reported estimates. However, our study only reports on children up to the age of 5. For a true comparison of our incidence to prior studies we need previously reported fracture incidence specific to this age group. One epidemiologic study, which only included inpatient and emergency room data, from the US reported an annual incidence of fracture of 4.38 per 1000 children in children less than 5 years old [10]. Two additional studies out of Sweden and Wales which examined both inpatient and outpatient data found annual fractures incidences of 11 and 14.2 per 1000 children, respectively [2, 3]. Overall, given the universal nature of the health insurance benefit in the Military Health System and the ability to track the population from birth to 5 years, the fracture incidence found in our study is likely a reasonably accurate representation of the true occurrence and is consistent with previously reported values [2, 3].

Age has been shown across multiple studies to be a strong risk factor for fracture, with increasing age leading to increasing rate of fracture. In fact, the highest reported incidence is usually seen in the 10–14 year-old age group [10–13]. Due to the large number of patients in our study we were able examine the relationship between age and fracture rate by examining the incidence of fracture across the same patients throughout the first 5 years of their life. We also found that age was strong risk factor for fracture with our incidence increasing almost 5-fold from the less than one-year-old age group to the four-year-old age group. Since bone mineral density has been shown to increase with age throughout childhood, this increased rate of fracture is likely related to a combination of increased physical activity, sports participation, and other behavioral changes [14].

The most common fractures in children are forearm fractures which account for 25–43% of all childhood fractures [2–4, 15, 16]. Fractures of the upper extremity predominate over lower extremity fracture and account for approximately two-thirds of all pediatric fractures. In our study, forearm fractures were by far the most common fracture and had the highest incidence in every age group. The next most common fracture varied by age group although some trends did emerge (Table 2). In the less-than-one-year-old age group clavicle fractures were the second most common and tibia fractures were much rarer. In the one- and two-year-old age groups there was an increase in tibia fractures which is likely related to the children beginning to walk. In the three- and four-year-old age groups upper extremity fractures were found to increase and represent the top three most common fracture variants in both age groups.

Male gender was found to be a risk factor for fracture in our study with male children experiencing a 12% increased fracture rate as compared to female children. These results are similar to prior studies with Naranjie et al.^9^ finding an 18% increased fracture incidence in male children among a group of similarly aged children and Lyons et al.^2^ discovering a 12% increase in fracture incidence in male children. The general consensus is that this increased rate of fracture is due to difference in sports participation and other risk-taking behavior more common in male children [10]. However, this is likely an oversimplification and the true cause is probably multifactorial in nature. Even when controlling for sports participation Stracciolini [17] found that male athletes were still twice as likely to sustain a fracture as females.

Geographic location has previously been shown to impact the incidence of fracture in pediatric patients [12]. When comparing various geographic locations within the United States we found a trend towards more fractures within the northern United States, however this trend was not significant. When comparing US patients living within the states to US patients living overseas, we found that US patients living overseas had a 40% higher rate of fracture. The reason for this increased fracture rate is unclear and has previously been debated. Moustaki [4] felt that the country to country variance in fracture incidence was likely due to one of or a combination of four different factors including number of exposures, changes in injury prevention strategies, differing nutritional factors, or unique patient biology. While our study does not definitively answer this question the fact that the increased fracture rate is observed with the cohort simply moving geographic location suggest that there are more contributors than just patient biology.

There are many studies that have shown in adults that patient race is an important factor in determining a patient’s fracture risk [18, 19]. There is far less information when examining the impact of race on pediatric fractures, however. A cohort of South African children, followed from birth until 20 years old, showed a 2-fold increase in fracture rate in White children when compared to Black children or children of mixed ancestry [20]. We had similar findings with White children having a 70% increased fracture rate when compared with Black children. While some of the increased risk is likely due to behavioral differences, genetics also are expected to play a key role as history of fracture in a family member and maternal bone mineral density have been shown to impact the incidence of fracture in the child [20].

The role of socioeconomic status on fracture incidence has historically been difficult to study as family income is not information that is typically gathered in medical records and thus surrogate measures are often used. This, however, is not the case within a military population as pay is based primarily on rank and the rank of the patient’s sponsor is easily determined. In our study we split our patients into four socioeconomic classes, with junior enlisted having the lowest income, senior officers having the highest and the other two falling in the middle. The junior enlisted group had the lowest fracture rate of any group, followed by the senior enlisted which was about 10 % higher and then the two officer groups which were about 20% higher. There are many possible explanations for this finding including differences in activity as youth sports and teams can be very expensive. Other potential explanations include parental age, and nutritional factors.

It is estimated that each year 42 out of every 1000 children will experience abuse or neglect [21]. In children who experience abuse the second most common presenting symptom is fracture, behind only skin lesions. The reported annual incidence of fracture in abused children varies greatly between studies but is typically between 10 and 50% [22, 23]. This rate is higher in younger children, as is the proportion of fractures related to abuse as opposed to accidental trauma. King et al. [24] found that over half of abuse-related fractures occurred in children less than 1 year old, and 80% occurred in children less than 3 years old. In our study of children less than 5 years old we had 35 abuse related fractures for an annual incidence .03 per 1000 children. The annual incidence was highest in children less than one-year-old at .08 fractures per 1000 children. Overall, our incidence of abuse-related fractures was lower than previously reported. One possible explanation for this is that some of the more prominent risk factors for abuse are less common within military families including income below the poverty line, unemployment, and illegal drug use [23]. Additionally, DoD funded research examining have shown a rate of child abuse and mistreatment that is half that of the civilian sector.

There are multiple strengths to this study. It includes a large number of pediatric patients across a nationally representative sample making it much more applicable to the general population then a study set in one urban area. It also follows the same patients for the first 5 years of life making it less susceptible to yearly variations in injury rates. This study does have several limitations. Although the diagnostic coding was done at the time of injury, as with all administrative data sets, it is still subject to either coding and specificity error.

## Conclusion

Pediatric fractures are an important cause of morbidity in young children. In order to adequately prevent and treat fractures it is important to understand the incidence of these injuries along with risk factors associated with sustaining them. This study demonstrated that living outside the United States, being male, being White, and being the child of an officer all increase the chance of sustaining a fracture. Further studies are still needed to determine why these risk factors exist and how to best modify them.

## Data Availability

All data generated or analyzed during this study are included in this published article.
